# Thiazide‐Induced Acute Interstitial Pancreatitis in a 55‐Year‐Old Male: A Rare Adverse Drug Reaction

**DOI:** 10.1002/ccr3.70813

**Published:** 2025-08-19

**Authors:** Kshitiz Pandey, Ashish Panday, Pratik Pandey, Anand Chaudhary, Sushmita Khanal

**Affiliations:** ^1^ Bakulahar Ratnanagar Hopsital Chitwan Nepal; ^2^ Tribhuvan University Teaching Hospital Kathmandu Nepal; ^3^ Nobel Medical College Teaching Hospital Biratnagar Nepal

**Keywords:** acute pancreatitis, drug‐induced pancreatitis, hypertension, interstitial pancreatitis, thiazide diuretics

## Abstract

Acute pancreatitis is inflammation of the pancreas and is most commonly caused by gallstones, alcohol use, hyperlipidemia, or idiopathic. Drug‐induced acute pancreatitis is relatively rare and often under‐recognized; it accounts for 0.1%–2% of all cases. Among many drugs, thiazide diuretics are infrequently implicated but remain a known cause. We report a case of a 55‐year‐old male who presented with acute epigastric pain, nausea, and vomiting 2 weeks after adding thiazide for persistent secondary hypertension. Laboratory investigations revealed elevated pancreatic enzyme levels, and contrast‐enhanced computed tomography confirmed acute interstitial pancreatitis. Comprehensive evaluation excluded common etiologies including gallstones (ultrasound, CT‐scan, and MRCP), alcohol use (history), and dyslipidemia (normal lipid profile). The temporal relationship between drug initiation and symptom onset made thiazide‐induced pancreatitis a likely diagnosis. Discontinuation of hydrochlorothiazide led to clinical improvement for the patient. This case highlights the importance of considering thiazide‐induced pancreatitis in patients who present with acute pancreatitis when the common risk factors are absent. While extensive investigation to search for other rare causes of acute pancreatitis should be done, prompt recognition and withdrawal of the possible offending agent are essential for patient recovery and prevention of recurrence.

**Summary**

- Thiazide diuretics, though rare can induce acute pancreatitis, especially when common etiologies are exclude.
- Clinicians should maintain a high degree of suspicion for drug‐induced pancreatitis in new‐onset cases and discontinue the suspected agent to help in recovery and prevent recurrence.

## 1 Introduction

Acute pancreatitis is a potentially life‐threatening inflammatory condition of the pancreas with a wide spectrum of clinical presentations. The most common etiologies include gallstones, chronic alcohol consumption, hypertriglyceridemia, certain infections, and idiopathic causes. However, drug‐induced pancreatitis remains a less frequent but important and often under‐recognized cause, accounting for approximately 0.1%–2% of cases [1, 2, 3]. The World Health Organization (WHO) has identified over 500 medications as potential causes of acute pancreatitis as an adverse drug reaction [4]. Most of the cases of drug‐induced pancreatitis cause mild to moderate pancreatitis and recover with fewer complications following the withdrawal of offending drugs.

Among them, thiazide diuretics, widely prescribed for hypertension and heart failure, have been associated in many literatures [1, 2, 3]. Although the exact pathophysiology is still unclear, proposed mechanisms include hypersensitivity reactions, drug‐induced hypercalcemia, and direct pancreatic toxicity [1, 3].

This case report highlights the importance of considering thiazide as a potential cause of acute interstitial pancreatitis in a patient with a recent start of intake, when there are no other identifiable etiological factors. The temporal relationship between drug initiation and symptom onset should be considered when diagnosing drug‐induced acute pancreatitis.

## 2 Case Presentation

### 2.1 Presenting Complaints and Disease History

A 55‐year‐old Asian man visited the outpatient department (OPD) for a routine check‐up on March 10, 2025. He was hypertensive for 5 years for which he was taking losartan 50 mg daily. He was nondiabetic and had a family history of hypertension in his father (85 years) and brother (52 years). He was a nonsmoker, nonalcoholic, and followed a vegetarian diet.

He complained of occasional episodes of mild diffuse headache. On examination, his blood pressure was 150/90 mm‐Hg in both arms, and other vital signs were within normal limits. Workup for secondary hypertension was done without significant findings and he was advised to follow lifestyle modifications, including regular exercise and the DASH (Dietary Approaches to Stop Hypertension) diet. He was also asked to maintain a blood pressure record at home for 1 week and return for follow‐up.

After 1 week, his home‐recorded blood pressure showed persistent elevation above 150/90 mmHg. A combination antihypertensive therapy**—**losartan 50 mg with hydrochlorothiazide 25 mg—was prescribed once daily in the evening, and he was advised to continue monitoring his blood pressure and return in 2 weeks.

Two weeks later, he presented to the emergency department with severe epigastric pain, nausea, and two episodes of vomiting. The pain was severe and constant, radiating to the back at **mid**‐thoracic region.

### 2.2 Investigations

**FIGURE 1:**
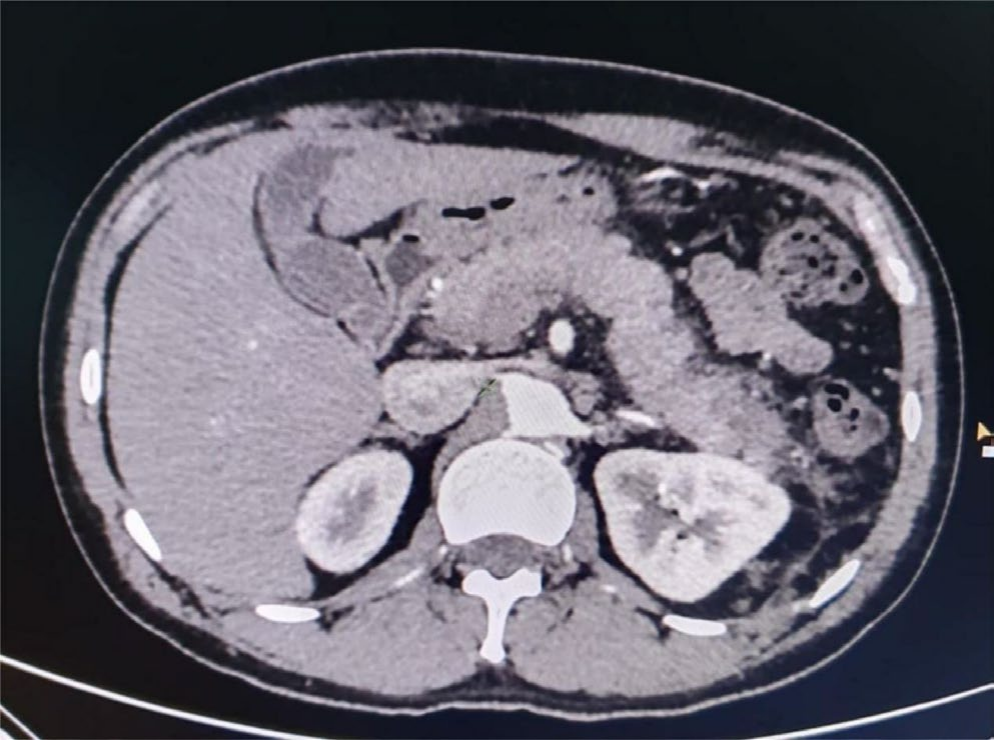
Acute interstitial pancreatitis with bulky pancreas, fuzzy margins, peri‐pancreatic fat stranding, and minimal free fluid.

Laboratory tests showed **l**eukocytosis with neutrophilia, elevated serum amylase and lipase levels with a normal lipid profile (Table 1). The pancreas could not be clearly visualized on the abdominal ultrasonogram and showed no gallstones. A contrast‐enhanced CT scan further confirmed the diagnosis of acute interstitial pancreatitis with a bulky pancreas, fuzzy margins, and minimal peripancreatic fluid collection with peripancreatic fat stranding (Figure 1). There was no necrosis, thrombosis, and pancreatic duct dilatation. The gallbladder appeared normal, and there was no dilation of the common bile duct (CBD).

**TABLE 1:** Laboratory values of patient on day of admission and 2 days after admission.

Parameters	Day of admission	Two days after admission	Reference range
Hemoglobin (Hb)	14.7 g/dL	13.5 g/dL	13.5–17.5 g/dL
Total WBC count	14,000/mm^3^	10,200/mm^3^	4000–11,000/mm^3^
Neutrophils	85%	72%	40%–70%
Lymphocytes	9%	20%	20%–40%
Platelet count	187,000/mm^3^	199,000/mm^3^	150,000–450,000/mm^3^
Lipid profile			
Total cholesterol	170 mg/dL	165 mg/dL	< 200 mg/dL
LDL cholesterol	95 mg/dL	90 mg/dL	< 130 mg/dL
HDL cholesterol	55 mg/dL	57 mg/dL	> 40 mg/dL (male)
Triglycerides	130 mg/dL	120 mg/dL	< 150 mg/dL
Serum calcium	9.2 mg/dL	9.3 mg/dL	8.6–10.2 mg/dL
Serum amylase	990 U/L	245 U/L	30–110 U/L
Serum lipase	1173 U/L	310 U/L	13–60 U/L
Electrolytes			
Sodium	139 mmol/L	141 mmol/L	135–145 mmol/L
Potassium	4.2 mmol/L	4.1 mmol/L	3.5–5.0 mmol/L
Chloride	101 mmol/L	100 mmol/L	98–107 mmol/L
Bicarbonate	23 mmol/L	24 mmol/L	22–28 mmol/L
Liver function tests (LFTs)			
Total bilirubin	0.8 mg/dL	0.7 mg/dL	0.3–1.2 mg/dL
Direct bilirubin	0.2 mg/dL	0.2 mg/dL	0.0–0.3 mg/dL
AST (SGOT)	32 U/L	30 U/L	10–40 U/L
ALT (SGPT)	28 U/L	26 U/L	7–56 U/L
ALP (Alkaline phosphatase)	88 U/L	85 U/L	44–147 U/L
Gamma GT	28 U/L		9–48 U/L
CRP	48 mg/L	22 mg/L	< 5 mg/L
Blood glucose (Fasting)	134 mg/dL	95 mg/dL	70–99 mg/dL

### 2.3 Differential Diagnosis/Exclusion of Common Etiologies

The absence of gallstones on imaging and a normal lipid profile excluded gallstones and hypertriglyceridemia. To verify the patient's claim of no history of alcohol intake, collateral history was taken from the patient's family, which was supported by a normal gamma‐glutamyl transferase (GGT) level of 28 U/L and undetectable blood alcohol concentration on admission. Imaging showed no features suggestive of chronic pancreatitis. These findings collectively helped us to exclude alcohol as an etiological factor in our patient. Autoimmune pancreatitis was excluded based on the absence of other autoimmune disorders, lack of involvement of other organs, and CT findings that were not consistent with the typical findings of autoimmune pancreatitis.

Blood cultures obtained at the time of presentation showed no growth after 72 h of incubation, excluding any bacterial infections. The patient resides in a non‐endemic area for scorpions, and there were no physical signs of a scorpion bite in a thorough physical examination. There was no history of recent travels, trauma, or surgical interventions.

### 2.4 Treatment

The patient was managed conservatively with **i**ntravenous fluids, analgesics, bowel rest (NPO). Hydrochlorothiazide was suspected as the potential cause of the pancreatitis and was discontinued. The patient showed gradual clinical improvement. He was discharged after 5 days of hospital stay with oral medications and advised to follow‐up in 2 weeks for further evaluation in search of etiology. An MRCP (Magnetic Resonance Cholangiopancreatography) was performed and found to be normal, ruling out biliary obstruction or pancreatic duct anomalies.

The combination of losartan and hydrochlorothiazide was permanently discontinued, and he was switched to a combination of losartan 50 mg and amlodipine 5 mg.

## 3 Discussion

Thiazide diuretics are the first‐line pharmacologic management of hypertension and are among the commonly prescribed antihypertensives [5, 6]. However, rare but serious adverse effects such as acute pancreatitis must be considered when more common etiologies have been ruled out. Our case highlights a case of drug‐induced acute pancreatitis in a patient initiated on thiazide therapy for hypertension.

Thiazide diuretics exert their antihypertensive effect by inhibiting sodium‐chloride symporters in the distal convoluted tubule, thereby reducing plasma volume and vascular resistance. Though the exact mechanism is still unclear, thiazide‐induced pancreatitis has been attributed to several potential mechanisms, including drug‐induced hypercalcemia, hypersensitivity reactions, pancreatic duct constriction, and direct cytotoxic effects on acinar cells [1, 7].

Diagnosis of acute pancreatitis is classically based on meeting two out of the following three criteria: Characteristic abdominal pain; serum lipase or amylase levels ≥ 3 times the upper limit of normal; and radiologic findings consistent with pancreatic inflammation [7]. Our patient met all three criterias. Finding the etiology of acute pancreatitis is a challenge when the common etiologies such as gallstones, alcohol use, hypertriglyceridemia, and trauma are excluded. Imaging in our case ruled out gallstone disease and laboratory evaluation did not reveal hyperlipidemia or hypercalcemia. No history of alcohol intake, infection, or prior abdominal trauma prompted the search for the rarer cause of acute pancreatitis. Thus, the recently initiated thiazide diuretic became the prime suspect.

Although drug‐induced acute pancreatitis accounts for a small proportion of cases (0.1%–2%), there is a huge list of drugs that has been linked with acute pancreatitis [8]. Among them, diuretics, mainly loop diuretics and thiazides, have been reported in many literatures [1, 2, 3, 4]. The European Case–Control Study on Drug‐Induced Acute Pancreatitis (EDIP) did not find a significant association between thiazide diuretic use and an increased risk of acute pancreatitis, suggesting that while the risk exists, it may be modest [9]. The case–control analysis by Ksiądzyna [10] showed that patients on diuretics present two and a half times more frequently with AP than the control group. Pravallika et al. [11] reported eight hydrochlorothiazide‐induced cases out of 31 cases (25.8%) of drug‐induced acute pancreatitis. The latency period between drug initiation and symptom onset may range from days to years, complicating recognition [1].

Patients at higher risk for developing thiazide‐induced pancreatitis include those with certain demographic and clinical characteristics. According to the analysis of the FDA Adverse Event Reporting System (FAERS), males and individuals aged 41–54 years are at increased risk for drug‐related acute pancreatitis, which includes thiazide diuretics. Additionally, patients with multiple comorbidities, such as hypertension, who are on polypharmacy regimens, may also be at higher risk due to the potential for drug interactions and cumulative adverse effects [12, 13].

Various pathophysiological mechanisms for thiazide‐induced pancreatitis have been proposed, including direct damage to pancreatic acinar cells which produce digestive enzymes, immune‐mediated pancreatic injury, pancreatic duct constriction possibly resulting from drug‐induced changes in smooth muscle tone or ductal pressure, and metabolic complications like thiazide‐induced hypercalcemia or hypertriglyceridemia, both are known risk factors for acute pancreatitis [1, 3, 6, 7, 8, 14]. Additionally, thiazides may cause ischemia of the pancreas by inducing hypovolemia and reducing pancreatic blood flow, which can contribute to pancreatic injury [13]. There is currently no specific genetic predisposition directly linked to the development of thiazide‐induced pancreatitis [9, 15]. Checking normal IgG4 levels could help exclude the possibility of acute episodes of autoimmune pancreatitis [16]. In our case, the patient had a normal lipid profile and calcium levels. Also, the electrolytes level was normal. Normal ductal luminal diameters of the biliary tree excluded pancreatic duct constriction. Thus, a hypersensitivity reaction or direct cytotoxicity is suspected based on laboratory findings and radiographical findings.

The cornerstone of initial therapy was aggressive intravenous fluid resuscitation aimed at counteracting third‐space fluid loss, maintaining pancreatic perfusion, and minimizing the risk of necrosis [8, 17]. Supportive measures including pain control with opioids and bowel rest were instituted; the suspected causative agent, that is, the thiazide diuretic, was immediately discontinued. Our patient improved clinically within 72 h with marked reduction in the level of pancreatic enzymes and resolution of symptoms.

Though there are no exact criteria to confirm a drug caused pancreatitis, clinicians need to consider the temporal association between drug exposure and symptom onset, rule out other possible causes, improvement after stopping the drug, and note if symptoms return when restarting the drug (usually not tested due to ethical concerns). Since thiazide‐related pancreatitis can develop long after starting the drug, it is also crucial to take a detailed medication history in any patient with pancreatitis of unknown cause. For those with ongoing issues like high calcium levels caused by thiazides, stopping the drug might be wise even before pancreatitis develops as a preventive measure.

## 4 Conclusion

Thiazide‐induced acute interstitial pancreatitis should be considered as a differential diagnosis of patients presenting with acute pancreatitis, and common causes such as gallstones, alcohol use, and metabolic disorders should be excluded with extensive investigations. Drug‐induced acute pancreatitis is a condition of exclusion. In this case, a strong temporal association between the drug initiation and symptom onset, along with resolution following drug withdrawal and absence of common causes, supports the diagnosis. Early recognition and discontinuation of the offending agent are crucial for optimal outcomes and prevention of recurrence. Patients education on such events to prevent future initiation of the offending drug should be focused.

## Author Contributions

**Kshitiz Pandey:** conceptualization, formal analysis, methodology, project administration. **Ashish Panday:** supervision, writing – original draft. **Pratik Pandey:** formal analysis, methodology, validation. **Anand Chaudhary:** project administration, resources, software, writing – review and editing. **Sushmita Khanal:** data curation, investigation, validation, writing – review and editing.

## Consent

Written informed consent was obtained from the patient to publish this report in accordance with the journal's patient consent policy.

## Conflicts of Interest

The authors declare no conflicts of interest.

## Data Availability

All the data underlying the results are available as part of the article and no additional source of data are required.

## References

[1] M. R. Jones , O. M. Hall , A. M. Kaye , and A. D. Kaye , “Drug‐Induced Acute Pancreatitis: A Review,” Ochsner Journal 15, no. 1 (2015): 45–51. 25829880 PMC4365846

[2] N. Chaaban and S. Kshatriya , “Thiazide ‐Induced Pancreatitis, Case Report,” Kansas Journal of Medicine 15, no. 2 (2022): 220–221. 35762001 10.17161/kjm.vol15.16534 PMC9225032

[3] T. Kaurich , “Drug‐Induced Acute Pancreatitis,” Baylor University Medical Center Proceedings 21, no. 1 (2008): 77–81. 18209761 10.1080/08998280.2008.11928366 PMC2190558

[4] C. M. Drug , “Drug‐Induced Acute Pancreatitis,” cited Apr 25, 2025, http://pancreapedia.org/?q=node/9038.

[5] R. H. G. Olde Engberink , W. J. Frenkel , B. van den Bogaard , L. M. Brewster , L. Vogt , and B. J. H. van den Born , “Effects of Thiazide‐Type and Thiazide‐Like Diuretics on Cardiovascular Events and Mortality: Systematic Review and Meta‐Analysis,” Hypertension 65, no. 5 (2015): 1033–1040. 25733241 10.1161/HYPERTENSIONAHA.114.05122

[6] G. Mancia , R. Fagard , K. Narkiewicz , et al., “2013 ESH/ESC Guidelines for the Management of Arterial Hypertension: The Task Force for the Management of Arterial Hypertension of the European Society of Hypertension (ESH) and of the European Society of Cardiology (ESC),” Journal of Hypertension 31, no. 7 (2013): 1281–1357. 23817082 10.1097/01.hjh.0000431740.32696.cc

[7] J. Saini , D. Marino , N. Badalov , M. Vugelman , and S. Tenner , “Drug Induced Acute Pancreatitis: An Evidence Based Classification (Revised),” Clinical and Translational Gastroenterology 14, no. 8 (2023): e00621, 10.14309/ctg.0000000000000621. 37440319 PMC10461957

[8] T. W. Underwood and C. B. Frye , “Drug‐Induced Pancreatitis,” Clinical Pharmacy 12, no. 6 (1993): 440–448. 8403815

[9] I. A. Eland , A. Sundström , G. P. Velo , et al., “Antihypertensive Medication and the Risk of Acute Pancreatitis: The European Case‐Control Study on Drug‐Induced Acute Pancreatitis (EDIP),” Scandinavian Journal of Gastroenterology 41, no. 12 (2006): 1484–1490. 17101581 10.1080/00365520600761676

[10] D. Ksiądzyna , “Drug‐Induced Acute Pancreatitis Related to Medications Commonly Used in Gastroenterology,” European Journal of Internal Medicine 22, no. 1 (2011): 20–25. 21238888 10.1016/j.ejim.2010.09.004

[11] P. Chadalavada , C. R. Simons‐Linares , and P. Chahal , “Drug‐Induced Acute Pancreatitis: Prevalence, Causative Agents, and Outcomes,” Pancreatology 20, no. 7 (2020): 1281–1286. 32878711 10.1016/j.pan.2020.07.401

[12] L. Zhang , W. Mao , D. Liu , et al., “Risk Factors for Drug‐Related Acute Pancreatitis: An Analysis of the FDA Adverse Event Reporting System (FAERS),” Frontiers in Pharmacology 17, no. 14 (2023): 1231320. 10.3389/fphar.2023.1231320 PMC10690789 38044938

[13] C. D. Trivedi and C. S. Pitchumoni , “Drug‐Induced Pancreatitis: An Update,” Journal of Clinical Gastroenterology 39, no. 8 (2005): 709–716. 16082282 10.1097/01.mcg.0000173929.60115.b4

[14] G. Goodchild , M. Chouhan , and G. J. Johnson , “Practical Guide to the Management of Acute Pancreatitis,” Frontline Gastroenterology 10, no. 3 (2019): 292–299. 31288253 10.1136/flgastro-2018-101102 PMC6583768

[15] D. C. Whitcomb , “Genetic Aspects of Pancreatitis,” Annual Review of Medicine 61, no. 1 (2010): 413–424. 10.1146/annurev.med.041608.121416 20059346

[16] R. Pezzilli , “Acute Recurrent Pancreatitis: An Autoimmune Disease?,” World Journal of Gastroenterology 14, no. 7 (2008): 999–1006. 18286678 10.3748/wjg.14.999 PMC2689427

[17] S. Tenner , S. S. Vege , S. G. Sheth , et al., “American College of Gastroenterology Guidelines: Management of Acute Pancreatitis,” American Journal of Gastroenterology 119, no. 3 (2024): 419–437. 38857482 10.14309/ajg.0000000000002645 PMC13221274

